# A pilot study to determine the feasibility of enhancing cognitive abilities in children with sensory processing dysfunction

**DOI:** 10.1371/journal.pone.0172616

**Published:** 2017-04-05

**Authors:** Joaquin A. Anguera, Anne N. Brandes-Aitken, Ashley D. Antovich, Camarin E. Rolle, Shivani S. Desai, Elysa J. Marco

**Affiliations:** 1 Department of Neurology, University of California, San Francisco, United States of America; 2 Department of Psychiatry, University of California, San Francisco, United States of America; 3 Department of Pediatrics, University of California, San Francisco, United States of America; TNO, NETHERLANDS

## Abstract

Children with Sensory Processing Dysfunction (SPD) experience incoming information in atypical, distracting ways. Qualitative challenges with attention have been reported in these children, but such difficulties have not been quantified using either behavioral or functional neuroimaging methods. Furthermore, the efficacy of evidence-based cognitive control interventions aimed at enhancing attention in this group has not been tested. Here we present work aimed at characterizing and enhancing attentional abilities for children with SPD. A sample of 38 SPD and 25 typically developing children were tested on behavioral, neural, and parental measures of attention before and after a 4-week iPad-based at-home cognitive remediation program. At baseline, 54% of children with SPD met or exceeded criteria on a parent report measure for inattention/hyperactivity. Significant deficits involving sustained attention, selective attention and goal management were observed only in the subset of SPD children with parent-reported inattention. This subset of children also showed reduced midline frontal theta activity, an electroencephalographic measure of attention. Following the cognitive intervention, only the SPD children with inattention/hyperactivity showed both improvements in midline frontal theta activity and on a parental report of inattention. Notably, 33% of these individuals no longer met the clinical cut-off for inattention, with the parent-reported improvements persisting for 9 months. These findings support the benefit of a targeted attention intervention for a subset of children with SPD, while simultaneously highlighting the importance of having a multifaceted assessment for individuals with neurodevelopmental conditions to optimally personalize treatment.

## Introduction

Five percent of all children suffer from Sensory Processing Dysfunction (SPD)[1], with these individuals exhibiting exaggerated aversive, withdrawal, or seeking behaviors associated with sensory inputs [2]. These sensory processing differences can have significant and lifelong consequences for learning and social abilities, and are often shared by children who meet Diagnostic and Statistical Manual-Fifth Edition (DSM-5; [3]) criteria for conditions such as Autism Spectrum Disorder and Attention Deficit/Hyperactivity Disorder (ADHD; [4–6]). Although there is symptom overlap, children with SPD often fail to receive services despite having similar behavioral impairments. This disparity of treatment in this population highlights the benefits of using quantitative assessments to determine one’s specific needs as well as the most beneficial type of intervention, versus the use of behavior-based labels to guide treatment.

While the label of SPD is based on behavioral observation of atypical response to sensory input, it is clinically recognized that many of these children also show challenges with attention. Conversely, convenience samples of clinically referred children with ADHD estimate that between 46–69% also show symptoms of sensory over-responsivity [7,8]. Characterization of the comorbidity between attentional abilities and sensory abnormalities in children often rely upon parent report questionnaires for ease of administration [6–9]. Recent work has demonstrated that some children with SPD have measurable structural differences in white matter tracts that correlate with attention abilities [10]. However, to our knowledge, no studies have performed direct detailed assessments of cognitive control (defined here as attention, working memory, and goal management, or multitasking [11,12]) in this population using either behavioral measures or functional neuroimaging. These types of characterizations are essential for subsequent development of cognitive remediation efforts that are guided by data-driven mechanistic targets.

The first goal of this study was to quantitatively evaluate attentional abilities in a cohort of children with SPD (and typically developing controls) using direct behavioral and neural measurements as well as parental reports of inattention. We used three computerized tasks to probe sustained attention, selective attention, and goal management abilities. In addition, we used midline frontal theta power, a known electroencephalography (EEG) marker of attentional abilities captured in real-time [13–15], as our neural metric of attention. Our second goal was to assess how each group responded to an at-home self-paced cognitive training intervention targeting attentional abilities. Similar approaches aimed at improving attentional deficiencies have shown considerable promise [12], including training in pediatric populations [16–23]. Given that attention deficits, defined broadly, result from numerous etiologies and exist in varying degrees of severity [24–26], it is likely that individuals identified a priori to have a specific attentional challenge would benefit most from training that aspect of cognitive control. We hypothesized that *(i)* children with SPD would show deficits in selective attention compared to their typically developing control (TDC) counterparts; *(ii)* children with SPD would show reduced midline frontal theta activity relative to TDC participants, and *(iii)* children with comorbid SPD and parent-reported attention concerns would show greater benefit from attention enhancing efforts than those without attention concerns.

To test these intervention-based hypotheses, we administered the aforementioned behavioral and neural measures of attention as well as a standardized clinical parent report measure for ADHD, the Vanderbilt ADHD Diagnostic Parent Rating Scale [27], prior to and following the attention-focused intervention. The Vanderbilt test was used to delineate two sub-groups within our SPD cohort: those that exceeded the standardized cut score for inattention or hyperactivity (SPD_+IA_) and those that did not (SPD).

## Methods

### Participants & screening measures

**Fig 1:**
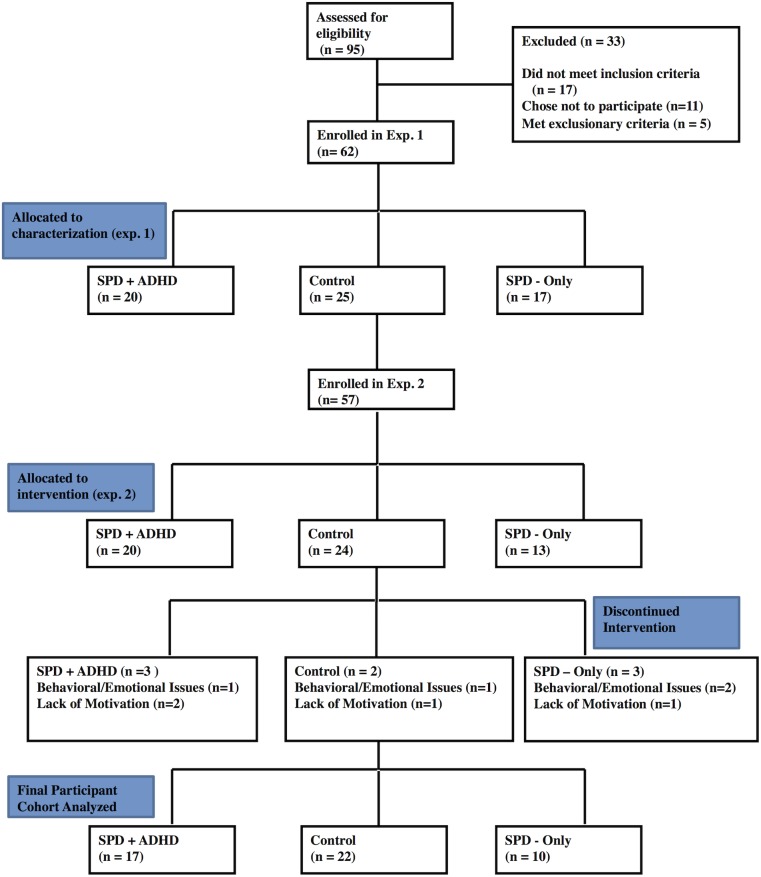
CONSORT flowchart for experiment 1 and experiment 2.

Participants were recruited from the UCSF Sensory Neurodevelopment and Autism Program (SNAP), participant registry and local online parent groups. For Experiment one, we recruited 20 children with SPD_+IA_ (8 female; age 9.7 +/- 1.3) age and gender matched with 17 children with SPD (8 female; age 10.3+/- 1.5), and 25 neurotypical children (12 female; age 10.5 +/- 1.3, see **Fig 1, S1 Checklist and S1 Protocol**). Participant recruitment began in February of 2014 and ended in January of 2015. This study was registered via the ISRCTN registry [ISRCTN #10912124] as a retrospective trial. This study was not registered before patient recruitment began as the study was conceptualized to be a characterization-based investigation of attention in children with neurodevelopmental conditions. Further, the second part of this study, the behavioral intervention, was implemented to gauge the feasibility and potential efficacy of using these types of approaches in these populations that could be used for a subsequent large-scale intervention trial. The authors confirm that all ongoing and related trials for this drug/intervention are registered. The UCSF Committee on Human Research IRB approved this study’s procedures on 01/02/2014. All parents provided written consent on behalf of their children, while children provided informed assent. UCSF’s IRB committee approved the consenting procedure used in this study. Exclusion criteria were brain malformation or injury, movement disorder, bipolar disorder, psychotic disorder, hearing impairment, or Perceptual Reasoning Index (PRI) score <70 on the Wechsler Intelligence Scale for Children–Fourth Edition [28].

All children were administered The Social Communication Questionnaire (SCQ; [29]. Any participant who scored above 15 points was administered the Autism Diagnostic Observation Schedule, Module 3 (ADOS; [30]) and excluded if they met ASD criteria on the ADOS. Two participants scored above SCQ threshold and were administered an ADOS in which they scored as non-spectrum and were subsequently included in our SPD_+IA_ group for analysis. One subject from the SPD_+IA_ group was excluded for scoring above 15 point on the SCQ and declining an ADOS assessment. Six children in our SPD_+IA_ cohort were on medication for ADHD symptoms and/or mood regulation, two children in the SPD group were on medications for ADHD symptoms and/or allergies, and 1 child in the TDC group was on medication for allergies. All children were prescribed stable dose prescribed medications for at least 6 weeks prior to initial assessment and initiation of the intervention (See **S1 File–**for participant medication information). Inclusion criteria for the SPD cohort was based on the most widely used sensory assessment, the Sensory Profile (a parent report questionnaire; [31]. All children in the SPD cohort had a community diagnosis of SPD and a score on the Sensory Profile in the “Definite Difference” range (<2% probability) in one or more of the sensory domains (auditory, visual, oral/olfactory, tactile, vestibular, or multisensory processing). None of the children in the TDC cohort scored in this range. Finally, the Vanderbilt Parent Report, a DSM IV based parent report, was administered to assess ADHD symptoms in our cohorts. See **S1 Table** for complete demographic information.

### Task descriptions

#### Perceptual discrimination paradigm

We administered a perceptual discrimination task derived from our previous work [13] to assess selective attention abilities. This task involved participants responding to specific stimuli presented on a computer monitor (green circles) while ignoring all other color/shape combinations. Participants were exposed to a 3 blocks of 36 target stimuli and 36 non-target stimuli, with each stimuli appearing on the screen for 400msec and an inter-trial interval of 2000-3000msec (with 500msec jitter). A fixation cross was present on the screen at all times above the car and below the appearing signs. Participants were instructed to look at the fixation cross at all times, and reminded of this after each run. The fixation cross provided the participant additional information to help their performance on each task: during the perceptual discrimination task, it turned green for 50msec when a relevant sign was responded to within the proper amount of time, or an irrelevant sign was ignored. When either of the aforementioned conditions were not met, it would turn red for 50msec indicating an incorrect trial.

#### Test of Variables of Attention (TOVA)

We administered the TOVA [32] to assess sustained attention and impulsivity abilities in our participants. With respect to the present study, the TOVA has demonstrated an estimated 85% sensitivity as a predictor of ADHD [33]. The TOVA is a 23-minute, fixed interval, visual continuous performance task administered on a laptop computer. Participants are instructed to respond a visual a visual stimuli (white square) appearing in the top edge (target stimuli) of the computer screen and to ignore the stimuli when it appeared at the bottom edge (non-target stimuli) of the computer. The stimulus appeared for 100ms every 2 seconds. The assessment is broken up into two parts measuring sustained attention (target stimuli appears in 22% of trails) and impulsivity (target stimuli appears in 77% of trials). Here we assessed response time and response time variability from the sustained condition in line with previous work using this measure in related populations.

#### Project: EVO™ (EVO) assessment and intervention

EVO is proprietary software developed by Akili Interactive Labs, specifically designed as a medical device to assess and adaptively target improvements in cognitive control for populations with cognitive disorders and executive function deficits. EVO was developed from the principles of a previous cognitive intervention known as NeuroRacer [13] but modified into an iOS mobile compatible application.

The EVO assessment is comprised of 3 tasks: perceptual discrimination, visuomotor tracking, and multitasking ability by performing each aforementioned task simultaneously. Critically, EVO incorporates adaptive psychometric staircase algorithms to ensure that comparisons between individuals reflect actual differences and not testing-based disparities. This approach also helps mitigate against any biases of age-related slowing, instrumentation, or ceiling/floor effects, finding an individualized level of performance that is specific to said user. Thus EVO changes its level of difficulty in a dynamic, trial-by-trial basis until the participant is performing at ~80% rate of accuracy [34–36]. The EVO assessment takes approximately seven minutes, with the primary variables associated with attentional abilities being mean response time and response time variability to target stimuli. Here we specifically focused on performance during the multitasking condition to avoid redundancy with our other attentional measures.

Unlike traditional assessments of attention, EVO was built to feel like a consumer grade videogame with a high interface environment and engaging visual and auditory feedback. An important feature of EVO is the adaptive algorithms embedded into its platform. Unlike most cognitive adaptive methods, EVO’s difficulty changes depending trial-by-trial performance. In addition, EVO provides the user with real-time feedback so the participant is consistently aware of their performance. The adaptive algorithm in EVO maximizes the specificity and sensitivity of the assessment by adjusting the difficulty of the game to keep the players accuracy around 80% accuracy. In the perceptual discrimination task, players complete a Go/No Go like-paradigm in which the user taps the iPad screen for correctly colored target stimuli while ignoring distracting targets. The visuomotor tracking task requires the participant to tilt the iPad to navigate their character through a dynamically moving road while avoiding walls and obstacles. The multitasking task requires participants to perform both perceptual discrimination and visuomotor tracking at the same time until participants complete a minimum number of trials and reach a stable level of performance.

The *Project*: *EVO*^*TM*^ intervention is a self-guided treatment designed for at-home use that involves a combination of visuomotor and perceptual discrimination tasks similar to those used during the EVO assessment. As previously mentioned, EVO was design in part on previous findings demonstrating that a custom-designed video game that pushes on cognitive control abilities, specifically attention and goal management, in the setting of interference can serve as a powerful tool for cognitive remediation [13]. Note that the use of the EVO intervention has recently shown beneficial effects on cognitive control abilities in other populations [37,38]. Each training run consists entirely of the multitasking condition, and lasts approximately 4 minutes, with 7 training runs comprising one day of training. As the participants improve their performance throughout this intervention, they are transported to different visual “worlds” in the EVO universe, meant to immerse the player and enhance the depth of engagement and compliance. Audio and visual cues are continuously available to the user so they are given feedback as to their performance. In addition, frequent EVO assessments are given to the player to obtain information as to how the player is improving throughout training, and adaptively set a personalized therapeutic regimen based specifically on the user’s own performance levels. The same adaptive mechanics utilized in the assessment are employed in the training sessions. Since the adaptive mechanics strive to keep the player at ~80% accuracy, the player is challenged to constantly improve upon their own cognitive control performance in order to reach the next level.

#### Basic response time task

We administered a measure of basic response time to ensure that any differences we see between groups are not due to differences in motoric quickness. In this task, participants respond to a target stimulus (40 trials) by tapping a button on an iPad platform. Similar to Project: EVO™, this task uses adaptive psychometric principles in a dynamic, trial-by-trial basis, with the primary variable of interest being one’s response time level (e.g. a fixed amount of time in msec that an individual is given to respond on a given trial) that allows for ~80% accuracy.

For Experiment 2, the EVO training regimen involved participant engagement with EVO 5 days a week for 1 month, with each day consisting of 7, 3–4 minute EVO sessions, with training occurring in the comfort of their own homes as opposed to a clinic or laboratory. Research assistants remotely monitored EVO play and provided support and feedback to the parents and children during training. If a research assistant noticed a participant had more than two incomplete days of training, a reminder phone call would be made to the parents. After a participant completed 20 days of EVO training, a follow-up research appointment was scheduled with the parents (see **S2 Table** more training-related information). All data, except for the EVO intervention, were collected at the University of California at San Francisco.

### Statistical analysis

All statistical analyses were conducted using SPSS 22.0 (SPSS Inc.). For experiment 1, group effects were assessed with separate ANOVAs with planned follow-up contrasts (Fisher’s LSD), with effect size for observed significant main effects reported with ETA^2^ (η2). Note that for this measure of effect size, .01 is considered to be a small effect, .06 = medium, .13 = large [39]. Follow up group contrasts (that is, when directly comparing one group to another) effect sizes are reported with Cohen’s d (calculated from estimated marginal means: Mean_1_-Mean_2_/Pooled SD, where .2 = small effect, .5 = medium, .8 = large [39]). For experiment 2, improvement in cognitive control was assessed with a linear mixed model repeated measures analysis, with subject acting as a random effect. For this analysis, a compound symmetry correlation structure among repeated measures was used to compare pre to post performance. Planned follow-up contrasts were constructed for each mixed model to directly assess changes within each group, with the effect sizes of these within-group changes reported via Cohen’s d. We also calculated the estimated marginal mean gain score (pre-post/post-pre) to further understand significant interactions from the mixed model analysis. To minimize influential data points, we removed values +/- 2 standard deviations for all group analyses. For correlations tested in Experiment 2, we used a more stringent outlier removal procedure (Cook’s D > 1 [40]) given the smaller cohort size and possible inflated change scores. De-identified individual scores for Experiment 1 and 2 are presented in the S1 Dataset.

## Results

### Experiment 1: Characterization of selective attention

#### Direct behavioral assessment of cognitive control

The first aim of this study was to characterize attentional abilities between the TDC (n = 25), SPD_+IA_ (n = 20) and SPD (n = 17) cohorts (for more details, see **S1 Table**) using three computerized tasks: the Test of Variables of Attention (TOVA [32]) probing sustained attention abilities; a perceptual discrimination assessment examining selective attention abilities [13]; and a video game-like assessment measuring goal management abilities (Project: EVO^TM^). We also administered a computerized measure of basic response time to assess for differences in motoric speed. Response time (RT) and response time variability (RTV) were the primary variables of interest for each test. The groups did not significantly differ in age (p = .16), gender (*χ*^2^ = .32, p = .85), nonverbal IQ (p = .48), or verbal IQ (p = .09), therefore we did not use any of these variable as covariate in any subsequent analyses.

The TOVA assessment revealed a main effect of group (F(2,51) = 6.3, p = .003, η2 = .20), with post-hoc tests indicating that the SPD_+IA_ group was significantly slower than the TDC group (p = .003, *d* = 1.2) and SPD group (p = .004, *d* = 1.0). The SPD cohort did not differ from the TDC group (p = .95, *d* = .02). RTV followed a similar pattern of results as above (F(2,52) = 6.3, p =. 004, η2 = .20), with post-hoc tests indicating that the SPD_+IA_ group had significantly greater RTV compared to the TDC (p =. 001, *d* = 1.1) and SPD group (p = .008, *d* = 1.0). The SPD group did not differ from the TDC group (p = .73, *d* = .10).

The perceptual discrimination assessment showed a significant effect of group (F(2,52) = 5.7, p = 0.006, η2 = .18), with the SPD_+IA_ group exhibiting significantly slower RT compared to TDC cohort (p = .002, *d* = 1.1). While the SPD_+IA_ cohort had modestly slower RT compared SPD children (p = .05, *d* = .75), the SPD group did not differ from the TDC group (p = .37, *d* = .31). Analysis of RTV revealed a significant effect (F(2,49) = 3.90, p = 0.027, η2 = .14), indicative of the SPD_+IA_ group being significantly more variable than the TDCs (p = .012, *d* = .80). The SPD cohort also showed a similar trend towards being significantly more variable than the TDC group (p = .06, *d* = .81), with no difference observed between the SPD_+IA_ and SPD groups (p = .61, *d* = .18).

**Fig 2:**
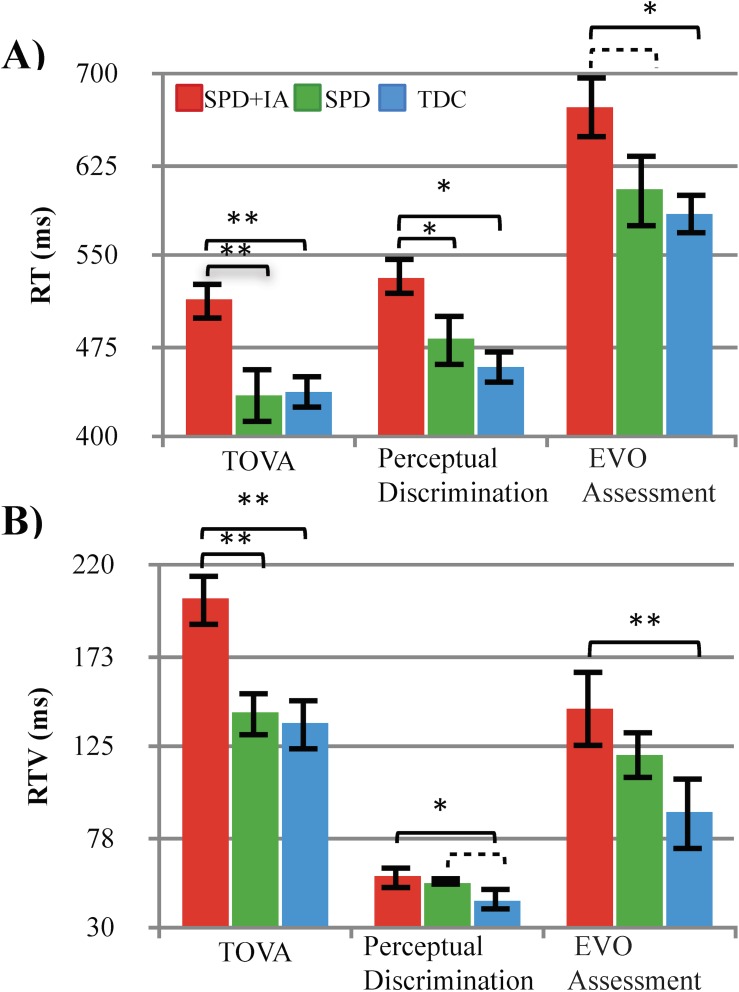
Comparison of performance on behavioral measures. (A) Response time and (B) response time variability revealing attention-related deficits in the SPD_+IA_ group compared to the SPD and TDC groups. Error bars indicate standard error of the mean. * = p≤ .05, ** =.p≤ .01, dashed brackets = p≤ .1.

Analysis of RT in the EVO assessment followed the same pattern as the results above (F(2,49) = 4.1, p = .023, η2 = .19, see **Fig 2**), with the SPD_+IA_ group being significantly slower than the TDCs (p = .007, *d* = 1.0). Furthermore, the SPD_+IA_ group showed a trend towards slower RT than the SPD group (p = .066, *d* = .65), but the SPD group showed no significant differences from the TDC cohorts (p = .55, *d* = .23). Similar effects were present for RTV (F(2,49) = 5.0, p = .01, η2 = .17), where the SPD_+IA_ was significantly more variable than the TDCs (p = .003, *d* = 1.0), but there was no difference between the SPD and SPD_+IA_ cohorts (p = .24, *d* = .35) or between the SPD and TDC groups (p = .11, *d* = .98).

There were no group differences present on the basic response time measure (F(2,41) = .57, p = .57, η2 = .03) or response time variability associated with this measure (F(2,40) = .22, p = .80, η2 = .01). This motor control measure suggests that the observed cognitive control group differences are not solely a function of motoric challenge.

#### Neural assessment of cognitive control

**Fig 3:**
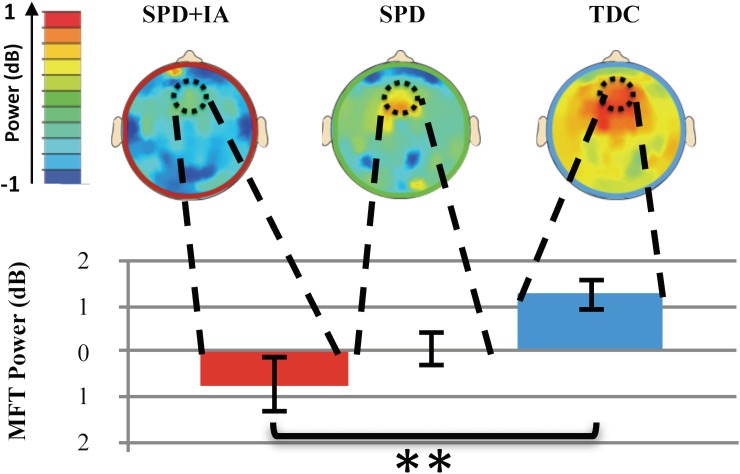
Comparison of neural activity during perceptual discrimination task. Bar graphs represent averaged MFT power between 280-320ms post target stimulus. Circles on heat maps present neural activity at the location and time of interest (4–7 Hz; 280-320ms). Error bars indicated standard error of the mean. * = p≤ .05).

We examined EEG recordings, specifically the midline frontal theta (MFT) rhythm, a well-established neural biomarker of attention, time-locked to the onset of stimulus during the performance of the perceptual discrimination task developed by Anguera and colleagues [13]; see **S2 File** for more details). We observed a significant group effect (F(2,45) = 4.2 p = .02, η2 = .16); see **Fig 3**). Post-hoc analyses revealed significantly lower MFT power for the SPD_+IA_ group compared to the TDC cohort (p = .006, *d* = .88), but no difference between the SPD_+IA_ and SPD cohorts (p = .31, *d* = .34) or between the SPD and TDC cohorts (p = .13, *d* = .78).

#### Experiment 1 summary

These results provide evidence that children with SPD and parental concern for inattention (SPD_+IA_) have measurably slower and more variable behavioral response, and weaker neural response on measures of attention relative to typically developing peers. The results from Experiment 1 informed our hypothesis for the following experiment.

### Experiment 2: Attention-based training

57 participants (SPD_+IA_ n = 20, SPD = 13, TDC n = 24; age-matched, p = .16) of the 62 individuals from Experiment 1 chose to participate in the second experiment. Experiment 2 aimed to determine if targeted attention remediation with a training version of Project: EVO™ (note that this version of EVO acts in a distinct manner from the EVO assessment platform; see **the methods section for more details**) would improve the identified selective attention, sustained attention, and goal management deficits in the SPD_+IA_ relative to the SPD and TDC cohorts (see **S2 Table** for details on participant attrition associated with training). We hypothesized that the SPD_+IA_ cohort, which had the greatest differences in direct behavioral assessment and neural measures, would show the greatest gains following the intervention. Training required participants to complete 7 rounds of EVO per day (~30 minutes), 5 days a week for 4 weeks (see **Methods** for more details). Participants were re-assessed on behavioral, neural and parent report measures at the completion of training (**see Fig 3A and 3B**) and parent report measures again at 9 months to query whether gains were enduring.

Prior to the intervention, participants self reported how often they engaged with recreational videogames. Chi-square analysis of this data revealed that frequency of videogame play did not differ between the groups (*χ*^2^ = 3.0, p = .86; **see Table 1**). Furthermore, when videogame play was converted to a continuous metric, the amount of time spent playing did not correlate with a change in any of the outcome measures (r*≤* .26, p≥ .19), suggesting that the following changes on these outcome measures do not appear to be associated with amount of videogame use outside of the assigned intervention.

**Table 1:** Frequency of Participant Videogame Play Per Week.

	SPD_+IA_	SPD	TDC
Less than 1 hour	17%	22%	14%
1 to 5 hours	58%	44%	67%
5 to 10 hours	25%	22%	14%
More than 10 hours	0%	11%	5%

#### Direct behavioral assessment of cognitive control: Post training

For the TOVA, there was no significant group x session interaction present for RT (F(2,43) = .345, p = .71), however a significant main effect of session was observed (F(1,44) = 19.05, p≤ .001). Similarly, although there was not a significant group x session interaction for RTV (F(2,46) = .005, p = .99), a main effect of session was present (F(1,47) = 5.4, p = .024), suggesting all groups improved on this measure following training.

For the Perceptual Discrimination Task, a trend towards a group x session interaction was observed on the perceptual discrimination assessment (F(2,40) = 2.7, p = .08) in addition to a significant main effect of RT (F(1,40) = 26.5, p<. 001). Post hoc analyses revealed that both the SPD_+IA_ (avg. change = 68.2ms, p≤ .001, *d* = 1.0) and the TDC (avg. change = 35ms, p = .005, *d* = .45) groups had improved response times, compared to the SPD group who did not show significant improvement (avg. change = 23.5ms, p = .17, *d* = .29). RTV, however, did not show a group x session interaction effect nor a main effect of session (F(1,41)≤ 1.0, p≥ .32).

**Fig 4:**
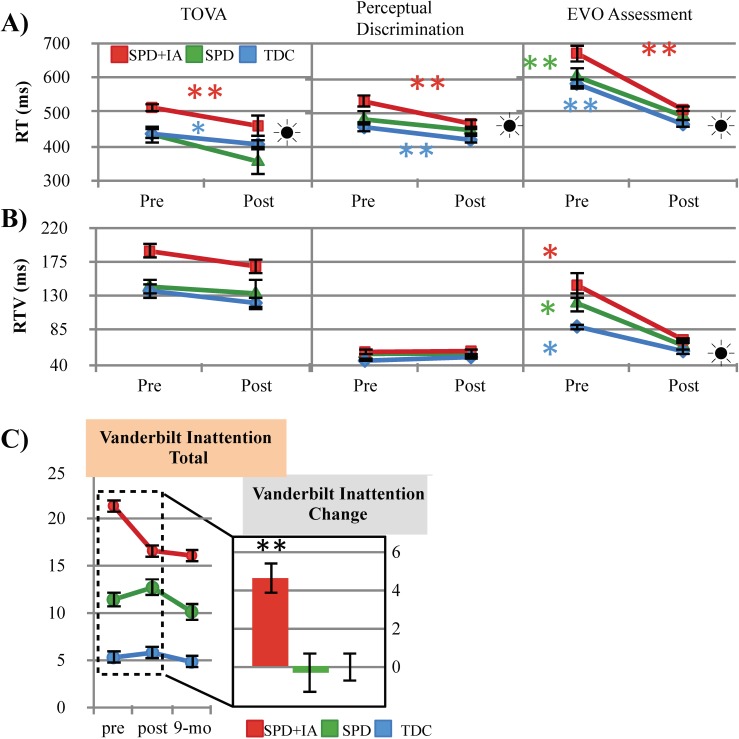
Transfer effect on behavioral and parent report measures. Pre and post (A) response time (B) and response time variability on behavioral tasks revealing within group change. Error bars indicate standard error of the mean. Within group main effects of session are designated as significant by stars: * = p≤ .05, ** =.p≤ .01. Sun symbols indicate statistically significant instances where SPD_+IA_ post-training performance was equivalent to or better than the TDC group prior to training. (C) Vanderbilt parent report inattention change bar plot (calculated by pre-post marginal means) and line plots revealing the significant group x session interaction. Error bars indicate standard error of the mean. All group x session interaction effects are designated as significant by stars (* = p≤ .05, ** =.p≤ .01) on bar graph.

RT in the EVO assessment did not show a group x session interaction effect (F(2,50) = 1.5, p = .24) but did reveal a significant main effect of session (F(1,50) = 103.3, p ≤.001). RTV also failed to show a group x session interaction (F(2,50) = 2.3, p = .11) but a main effect of session was present (F(1,50) = 45.3, p≤ .001, **see Fig 4A and 4B**), with these findings again suggesting that all groups improved with training on measures of both speed and reduced variability.

Analysis of the basic response time task did not show a significant group x session interaction (F(2,40) = .50 p = .62) or a significant main effect of session (F(1,41) = 1.6, p = .21). Similarly, RTV showed no significant session X group interaction (F(2,40) = .10 p = .90) nor a main effect of session (F(1,41) = .55, p = .46).

#### Parent report of attention: Post training & 9 month follow-up

To test whether the training led to changes in parent perceptions of inattention, we examined whether performance on the Vanderbilt parent report improved following training. This analysis revealed a significant group x session interaction (F(2,44) = 9.4, p≤ .001) and a significant main effect of session (F(1,44) = 7.1, p = .01). Post-hoc analyses revealed that only the SPD_+IA_ group showed significant decrease in parent observed inattentive behaviors (avg. change = 4.5 points, p≤ .001, *d* = 1.4). Importantly, these parent-reported improvements in inattentive behaviors remained stable for the SPD_+IA_ group at nine months post-training (p = .66, *d =* .14; see Fig 4C). Furthermore, 33% of individuals in the SPD_+IA_ group who initially met cut off for the Vanderbilt inattentive subtype no longer met criteria after training.

#### Neural assessment of cognitive control: Post training

**Fig 5:**
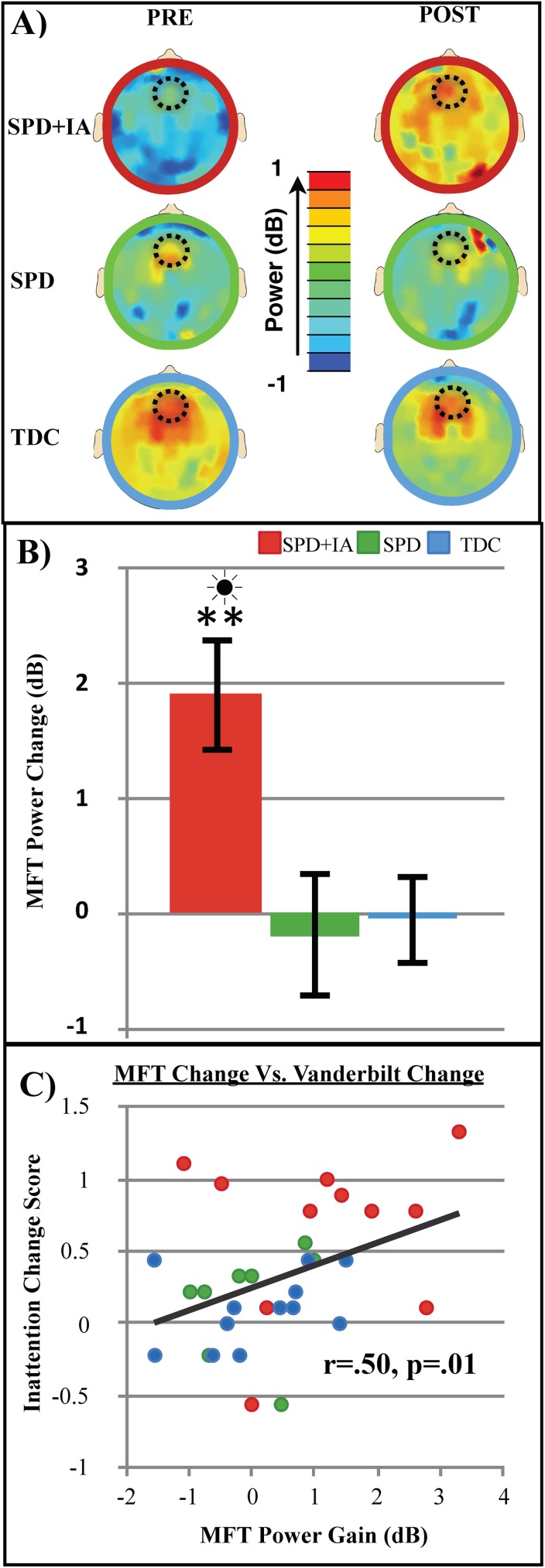
Transfer effects related to neural activity. (A) Neural midline frontal theta power pre and post training, (B) change in power (calculated using post-pre marginal means). Error bars indicate standard error of the mean. All group x session interaction effects are designated as significant by stars (* = p≤ .05, ** =.p≤ .01). Sun symbols indicate statistically significant instances where SPD+IA post-training performance was equivalent to or better than the TDC group prior to training. (C) Correlation between MFT power gain (post-pre) and Vanderbilt parent report change (pre-post) plots.

Midline frontal theta power changes following training revealed both a significant group x session interaction (F(2,30) = 5.9, p = .007) and a significant main effect of session (F(1,30) = 4.7, p = .04), with post hoc analyses indicating that the SPD_+IA_ group had a significant increase in MFT power following training (avg. change = 1.9_dB_, p≤ .001, *d* = 1.2; see Fig 5A and 5B).

We then performed a linear regression assessing baseline assessment response times for each task with change in the Vanderbilt parent report while controlling for cohort effects. This analysis revealed a significant association involving the change in the parent report with performance on both the TOVA (p = .037) and the perceptual discrimination task (p = .030; see **Table 2**), suggesting that baseline task performances could serve as a predictor for parent observed improvement with this cognitive training. Furthermore, we investigated whether there was a bivariate relationship between the behavioral change noted for individual children on the Vanderbilt inattention sub-scale and neural change in MFT power and found a positive correlation (r = .50 p = .01; see **Fig 5C**), suggesting that as MFT power improved, parent observed inattentive behaviors decreased.

**Table 2:** Multiple regression of baseline assessment response times in predicting Vanderbilt improvement.

	*P*	*Adj*. *R*^*2*^
Regression Model	0.001	.37
Group	.001	
TOVA	.037	
Perceptual Discrimination	.030	
EVO Assessment	.15	

#### Experiment 2: Summary

While all groups showed improvement on the behavioral measures of attention following training, only the SPD_+IA_ group showed differential neural and parent-reported benefits from attention remediation efforts, with improvements on these measures directly correlating with each other. Furthermore, when comparing the behavioral and neural outcomes of the SPD_+IA_ cohort following training to the TDC cohort at baseline, the SPD_+IA_ cohort’s was equivalent to (and in some cases better) than the TDC’s performance.

## Discussion

The present findings evidence attentional deficiencies in a subset (54%) of children with SPD, with these children showing the greatest gains in neural and parent-reported attention following a targeted-attention intervention. Thirty-three percent of these individuals no longer exceeded the clinical threshold for inattention, with parental report improvements persisting for 9 months. Here we discuss the utility of properly evaluating individuals using a multifaceted approach for tailoring remediation efforts in clinical populations, and the possible neural mechanisms underlying the parent observed behaviors.

### Behavioral markers of attention in children with SPD

To varying degrees, all children with SPD struggle to properly modulate incoming sensory information, making it difficult to function in the same way as their unaffected peers [1,41–43]. The observed behavioral and neural findings support the idea that a subset of the SPD population face greater cognitive control deficiencies compared to TDC, which acts as an additional impediment in their daily lives. This is of particular interest given that cognitive control abilities, specifically attention, have been shown to modulate sensory processing abilities [44–46]. Behaviorally, the variability of attentive behaviors in our SPD group supports the idea that there is a subset of SPD children who are less affected by attention deficits but still equally impeded by sensory dysfunction. Moreover, there is a segment of the SPD population who is not affected by ADHD symptomology, which provides evidence that SPD and ADHD are not synonymous conditions and these challenges must be assessed and considered independently in each individual [47].

The parent-reported improvement after training, accompanied by the persistent sustained effects 9 months later, provides support for targeted attention-based interventions having beneficial effects that can generalize for specific individuals. These findings agree with previous cognitive training work reporting similar effects in children with attention-based clinical symptoms [17,19–23], including the selective persistence of these attention-based benefits months later [13,16,17,19]. The training effects observed here are promising given that 33% of children who originally exceeded threshold scores for inattention/hyperactivity no longer met criteria after the intervention. It should also be noted that while only session main effects were present following training for each behavioral measure of attention, the SPD_+IA_ group performance either reached (TOVA, Perceptual Discrimination) or surpassed (EVO assessment) that of the TDC at baseline. Thus, these findings support the idea that those individuals with specific attentional deficits can benefit from interventions that selectively target these challenges [48–54].

### The neural basis for attention difficulties in children with SPD

A succinct explanation describing the most prevalent issue associated with SPD is that these individuals do not readily or effectively filter irrelevant sensory information. The observed impairments in performance and increased response variability across sustained attention, selective attention, and goal management measures suggests one possible unifying theory: children with inattention and SPD have disrupted cortico-thalamic connectivity. Inhibition-based dysfunction within the basal ganglia can impact both thalamic and prefrontal functions, hindering discrimination abilities that present in the form of poorly focused attention [55]. Impaired function along this circuit has been associated with attention based deficiencies in a multitude of conditions [56,57], including ADHD [58,59], with theta activity thought to relay communication between the basal ganglia and frontal regions [60]. Indeed, rodent models have demonstrated that thalamic inputs to the prefrontal cortex play the most crucial role in the observed alteration of information transmission [61].

This theory is supported by the improvement associated with midline frontal theta activity by the SPD_+IA_ cohort. Modulation of this neural marker following cognitive training has been previously demonstrated using a similar approach in older adults (NeuroRacer; [13]), and is suggested to reflect a reduction in one’s susceptibility to distraction [62].While deeper assessment of basal ganglia and thalamic connectivity in SPD children is warranted to confirm such ideas, recent neuroimaging work has demonstrated reduced white matter microstructural integrity in children with SPD being strongly correlated with inattention [10]. Thus, the present findings suggest that a thalamo-cortical dysfunction may underlie observed attentional deficiencies in this population, and a training platform that directly pushes on this impaired circuitry can remediate observed attention-based deficiencies. These findings support the need to explore whether distinct structural and functional networks underlie the observed cognitive control and sensory modulation challenges in children with sensory processing dysfunction.

### Limitations and conclusions

To our knowledge, this is the first study to quantitatively assess cognitive control abilities in an SPD cohort and attempt to remediate observed deficiencies using a behavioral intervention. These findings support the importance of a phenotypic-first characterization of each presenting individual to identify whether observed behavioral issues reflect deficient attentional abilities, abnormal sensory processing, or a combination of the two. The ability to first characterize and subsequently determine those individuals most likely to benefit from a specific targeted intervention directly addresses the idea of personalized medicine in this space [63].

While these findings are encouraging, further work is required to validate these initial results and address limitations present. For example, one could argue that the absence of differential group effects following training on the behavioral measures of attention could simply reflect practice-based improvements on these measures. However, this interpretation is unlikely given our previous work has used such measures to uncover training-related gains in attention [13]. A more likely explanation is that the present study may not have been sufficiently powered to dissociate attention-based improvements on these particular behavioral measures amongst the present populations using these tools. This result speaks to the utility of using a multiple assessments approach (e.g. behavioral, physiological, observer reports) to best understand the impact of any given intervention and ensure that a truly robust assessment is occurring in the spirit of personalized medicine. Another related limitation is the lack of a SPD placebo control group to better understand any training-related gains [11]. However, the use of an expectancy-matched placebo or no-contact control group for validation was precluded here for primarily practical reasons: this project represents a first step in determining the feasibility of enhancing cognitive abilities in this patient population, with the goal of identifying a potential signal of interest to subsequently perform a much larger mechanistic study with such a control group. These results provide empirical justification for future work that would involve differential control groups to truly understand the mechanisms at play here, including efforts that would be aimed at evidencing differential improvements on the outcome measures tested here. Such efforts would be bolstered by the incorporation of teacher reports of attention and other academic performance related outcomes to see the value of such training in more ‘real-world’ measures, as well as attempting to control participants’ use of consumer videogames, as regular practice on such platforms could theoretically affect one’s cognitive abilities.

The present findings also emphasize a switch to understanding neurodevelopmental disorders as a fluid and continuous set of symptoms rather than a discrete condition, as such an approach would support deeper characterization and more promising, targeted intervention outcomes. Being able to make such decisions based on quantitative measures, especially those that are palatable to the participants from a time and burden perspective, would remove any semblance of parental bias in these situations. However, one should not underestimate the functional utility and power of this particular parent report in characterizing a child’s basic cognition, given its observed relationship with midline frontal theta. Furthermore, the neural findings provide a possible mechanistic explanation for such improvements, providing an important neuroanatomical target for future work in this space. In summary, this research supports a shift towards a trait-based approach to best characterize attention and sensory dysfunction in children, and in doing so, customize intervention options to maximize real-world, sustained benefit.

## Supporting information

S1 Checklist TREND Statement Checklist. (PDF)

S1 Protocol EVO Training Protocol. Study protocol approved by UCSF Ethics Committee.
(DOCX)

S1 Table Demographic Information. Age, Handedness, IQ, and Ethnicity by group.
(DOCX)

S2 Table EVO Training Data. The number of days in training, training rounds completed, and diagnostic rounds passed by group.
(DOCX)

S1 File EEG Analysis. Pre-processing and statistical analyses protocols for the EEG data.
(DOCX)

S2 File Participant Medication Information. Overview of medication taken by the study participants.
(DOCX)

S1 Dataset Dataset. Data from Experiment #1 and #2 presented by group for each test reported.
(XLSX)
