# Supplementary material for: A pilot study to determine the feasibility of enhancing cognitive abilities in children with sensory processing dysfunction
Source: PLoS One. 2017 Apr 5;12(4):e0172616. doi: 10.1371/journal.pone.0172616 (PMC5381761; doi:10.1371/journal.pone.0172616)
Supplement: S1 Protocol — Study protocol approved by UCSF Ethics Committee. (DOCX) [file pone.0172616.s002.docx]

**Study Protocol Approved by Ethics Committee**

**Recruitment Criteria**

Neuropsychological and Cognitive Testing and Intervention Research:

1. Males and females, age 5-30 years
2. Children and adults diagnosed with a neurodevelopmental disorder or healthy children for control subjects.

3. Specific for Autism Imaging subjects ONLY:

          a. Autism Spectrum Disorder diagnosis according to DSM-IV-TR criteria; 
          b. Boys, age 8-11 years;

          c. IQ >70

4. Specific for Autism Adult Outcome subjects ONLY:

          a. Autism or PDD-NOS diagnosis according to DSM-IV-TR criteria;

          b. Male and Female Adults, age 21-30 years;

5. Specific for Sensory Processing Disorder subjects ONLY:

          a. Sensory Processing Disorder as observed through SensOR and Sensory Profile;

          b. Boys, age 8-11 years;

          c. IQ > 80

6. Specific for Attention Deficit Hyperactivity Disorder subjects ONLY:

          a. Autism Spectrum Disorder diagnosis according to DSM-IV-TR criteria;

          b. Boys, age 8-11 years;

          c. IQ > 70

**Procedures:**

Subjects will have the option to consent for multiple parts of the research, depending on eligibility.

*Cognitive testing may include IQ tests, executive function tests, social-emotion battery, the SPS (sensory processing scale developed by the Sensory Processing Disorder Foundation), and/or a semantics test battery. Some assessments may be done using a computerized tool.

| **Neuropsychological and Cognitive Testing and Intervention Research:** |
| --- |
| 1. Phone Screening for general inclusion/exclusion criteria |
| 2. Consents |
| 3. Diagnostic evaluation |
| 4. Parent-report questionnaires |
| 5. Cognitive and Neuropsychological Testing |
| 6. EEG Scan |
| 7. Computerized Intervention Assessment: The intervention aspect will be done outside of the UCSF vicinity, at the participant's home, over a 4 week time period. |

**Interviews, questionnaires, and/or surveys will be administered or focus groups will be conducted**

Dunn Sensory Profile

Behavior Assessment for Children, Second Edition (BASC-2)

Vanderbilt ADHD Diagnostic Parent Rating Scale

Autism Diagnostic Observation Schedule (ADOS)

Differential Screening Test for Processing (DSTP)

Sensory Integration and Praxis Test (SIPT)

Beery Visual Motor Integration (VMI)

Neuroracer*

Brain Baseline*

TOVA*

Evo*

*Computerized

**Time Commitment**

Phone Screening: 10 minutes

Consents: ½ hour (or more, if needed to ensure clarity and knowledgeable consent)

Diagnostic Evaluation: Evaluation of ASD takes about 3 hours and will commonly take place during a clinical assessment at the Autism Clinic at UCSF. Assessment of SPD using the SensOR takes 45 minutes.

Parent Questionnaires: Completion of all questionnaires will take approximately one hour and can be done at the parents' convenience. Families recruited through the Autism Clinic will complete the ALDI and PDDST-II as part of the clinical procedure, whether or not they elect to participate in research.

Cognitive Testing: Cognitive testing will require between 1-8 hours, depending on the number of cognitive tests the subject elects to participate in. IQ testing, the only test required to complete the brain imaging research, takes about one hour. These tests will take place over several visits.

EEG Scans: 1 hour each; 2 hours total

Intervention: 20 minutes each day for 5 days/week over a 4 week period
