# Supplementary material for: A pilot study to determine the feasibility of enhancing cognitive abilities in children with sensory processing dysfunction
Source: PLoS One. 2017 Apr 5;12(4):e0172616. doi: 10.1371/journal.pone.0172616 (PMC5381761; doi:10.1371/journal.pone.0172616)
Supplement: S1 Table — Age, Handedness, IQ, and Ethnicity by group. (DOCX) [file pone.0172616.s003.docx]

## S1 Table

|  | 20 SPD+IA (8 female) | 17 SPD (8 female) | 25 Controls (12 female) |
| --- | --- | --- | --- |
| **Age** | 9.7 +/- 1.3 (8.0-12.9) | 10.3 +/- 1.5 (8.0-12.5) | 10.5 +/- 1.3 (8.0-12.8) |
| **Handedness** |  |  |  |
| Right | 19 | 16 | 24 |
| Left | 1 | 1 | 1 |
| **IQ** |  |  |  |
| NVIQ | 111.2 +/- 16.0 | 110.3 +/- 16.0 | 115.3 +/- 11.0 |
| VIQ | 115.1 +/- 14.1 | 117.2 +/- 16.0 | 123.8 +/- 11.0 |
| **Ethnicity** |  |  |  |
| Caucasian | 13 | 11 | 14 |
| Asian | 0 | 0 | 2 |
| African American | 0 | 0 | 2 |
| Mixed Ethnicity | 6 | 6 | 2 |
| Unknown | 1 | 0 | 5 |

## Demographic Information
