# Supplementary material for: A pilot study to determine the feasibility of enhancing cognitive abilities in children with sensory processing dysfunction
Source: PLoS One. 2017 Apr 5;12(4):e0172616. doi: 10.1371/journal.pone.0172616 (PMC5381761; doi:10.1371/journal.pone.0172616)
Supplement: S2 Table — The number of days in training, training rounds completed, and diagnostic rounds passed by group. (DOCX) [file pone.0172616.s004.docx]

## S2 Table

**EVO Training Data**

|  | **SPD_+IA_** | **SPD_-IA_** | **Control** | **P-value** |
| --- | --- | --- | --- | --- |
| **Days in Training** | **31.4 (7)** | **30.7 (4)** | **36.3 (8.3)** | **F=3.2, p=.05** |
| **Training Rounds Completed** | **123.5 (14.3)** | **121.6 (27.1)** | **134.36 (21.5)** | **F=1.9, p=.16** |
| **Diagnostic Rounds Passed** | **14.5 (5.1)** | **14.4 (3.7)** | **14.5 (3)** | **F=.003, p=.99** |

Note: Data presented as mean (SD). Days in training refers to the to the average number of days it took participants to complete 20 training sessions (a session is comprised of 7 rounds), training rounds completed refers to the average number of training rounds a participant completed during the intervention period, completed diagnostic rounds refers to the average number of diagnostic rounds each participant completed and improved upon their prior diagnostic round performance.
