# Supplementary material for: A pilot study to determine the feasibility of enhancing cognitive abilities in children with sensory processing dysfunction
Source: PLoS One. 2017 Apr 5;12(4):e0172616. doi: 10.1371/journal.pone.0172616 (PMC5381761; doi:10.1371/journal.pone.0172616)
Supplement: S1 File — Pre-processing and statistical analyses protocols for the EEG data. (DOCX) [file pone.0172616.s005.docx]

## S1 Supporting Information

## EEG Analysis

While participants preformed the perceptual discrimination task, electroencephalography (EEG) activity was recorded with Active Two head cap (Cortech-Solutions) with a BiosSemi ActiveTwo 64-channel EEG acquisition system in conjunction with BioSemi ActiView software (Cortech-Solutions). Signals were amplified and digitized at 1024 Hz with a 16-bit resolution. Anti-aliasing filters were used and data were band-pass filtered between 0.01–100 Hz during data acquisition.

Data was preprocessed using Analyzer software (Brain Vision, LLC), with blinks and eye-movement artifacts removed through an independent components analysis, as were epochs with excessive peak-to-peak deflections (±100mV). These data were then exported into EEGLAB (61) for event-related spectral perturbation (ERSP) analysis. As in our previous work (12) epochs of -1000 to +1000 ms were created for ERSP total power analysis (evoked power + induced power), with theta band activity analyzed by resolving 4–100 Hz activity using a complex Morlet wavelet and referenced to a -900 to +700 pre-stimulus baseline (thus relative power (dB)).

## ERSP Statistical Analysis

To determine a MFT power difference between the SPD_+IA_, SPD, and TDC we first created 1 frontal composite electrodes of interest (EOI) from the average of AFz, Fz, FPz, AF3 and AF4 electrodes as in our previous work (12). ERSP data was segmented into 40ms time bins from 0-400ms where time 0 represents the onset of the target stimulus. Collapsing across groups, we looked for the time period where MFT power was maximal, revealing that peak MFT power occurred between 200-400ms. An initial ANCOVA revealed a significant effect of group on MFT power within this time window (F(1,47)=5.8, p= .02), with the SPD group showing significantly lower MFT power compared to their TDC counterparts. As 200msec represents approximately one full theta oscillation, we selected the median 40msec time bin from this time period (280-320msec) and used it for all subsequent analyses.
