# Supplementary material for: A pilot study to determine the feasibility of enhancing cognitive abilities in children with sensory processing dysfunction
Source: PLoS One. 2017 Apr 5;12(4):e0172616. doi: 10.1371/journal.pone.0172616 (PMC5381761; doi:10.1371/journal.pone.0172616)
Supplement: S2 File — Overview of medication taken by the study participants. (DOCX) [file pone.0172616.s006.docx]

# S2. Supporting Information

# Participant Medication Information

In the SPD_+IA_ group, 6 children were taking stimulant medications (methylphenidate, dexmethylphenidate, lisdexamfetamine) for ADHD symptoms. In addition to simulant medication, 3 of those children were taking a drug for mood regulation (SSRI or lamotrigine), and 2 of those children were being treated with guanfacine for hyperactivity. In the TDC group, 1 child was taking was taking oxybutynin for an over-active bladder and 1 child was taking Singulair for allergies. In the SPD group, one child was taking Vitamin D, Iron and Algea omega to help with attention and also flucticasone for allergies. Another child was taking a methylphenidate and guanfacine for ADHD symptoms. All children were on a stable dose of medication for 6 weeks prior to enrolling in the study.
